# Low vision and the risk of dementia: a nationwide population-based cohort study

**DOI:** 10.1038/s41598-020-66002-z

**Published:** 2020-06-04

**Authors:** Ji-Sun Paik, Minji Ha, Youn Hea Jung, Gee-Hyun Kim, Kyung-Do Han, Hyun-Seung Kim, Dong Hui Lim, Kyung-Sun Na

**Affiliations:** 1 Department Department of Ophthalmology, College of Medicine, The Catholic University of Korea, Seoul, Republic of Korea; 2 Department of Statistics and Actuarial Science, Soongsil University, Seoul, Republic of Korea; 3 Department of Ophthalmology, Sungkyunkwan University School of Medicine, Samsung Medical Center, Seoul, Republic of Korea

**Keywords:** Visual system, Dementia

## Abstract

Recent studies suggested that an association exists between vision loss and cognitive impairment, although it is still vague whether there are causal relationships or direct association between low vision and dementia. We were to investigate the association between low vision and dementia in the Korean population using the National Health Insurance Service (NHIS) database. We analyzed the data of 6,029,657 subjects aged ≥40 years, drawn from Korea National Health Insurance Service. The hazard ratio (HRs) and 95% confidence interval (CIs) of dementia, Alzheimer’s disease (AD), and Vascular dementia (VD) were estimated using multivariable Cox proportional hazards regression models. Statistical analysis showed that subjects with more severe visual impairments have a higher risk of dementia, AD, and VD after adjusting for compounding variables. The HRs of dementia increased significantly as visual acuity worsened (HRs 1.444 [95% CIs 1.415–1.473] for visual acuity (VA) < 1.0, 1.734 [1.693–1.777] for VA < 0.3, 1.727 [1.686–1.770] for VA < 0.1 and 1.991[1.902–2.085] for visual loss). Baseline visual loss and visual impairment were positively associated with the risk of dementia, AD, and VD. From the results of this nationwide population-based cohort study, we suggest that there is a significant increase in the incidence of dementia in subjects with low vision.

## Introduction

Dementia is a neurodegenerative disorder characterized by a progressive decline of memory and cognitive function^1–3^, and is recognized by the World Health Organization as a global public health priority^4^. Alzheimer’s disease (AD), which accounts for 60–80% of dementia, is the most common cause of dementia, followed by vascular dementia (VD)^2,3,5^. Current estimates suggest that 44 million people live with dementia worldwide, with global estimates predicted to quadruple by 2050 as the population ages^2,6–8^. The annual cost of dementia in the USA alone may exceed $600 billion, which is a large burden on the society^9,10^. Recent studies reported that the incidence of dementia in western countries may be declining whereas low and middle income countries are predicted to have the largest increase in incident dementia^8,11^. This discrepancy is suggested to be due to the differences in the effective management of cardiovascular disease, hypertension, and diabetes in these regions^8^. However, even though early recognition of risk factors for dementia is of utmost priority, the sporadic incidence of dementia is a challenge in preventive medicine^2,3^.

Previous studies have suggested that an association exists between vision loss and cognitive impairment^12–16^. Low vision and blindness are commonly seen in the older population as the risk of developing cataract; age-related macular degeneration and glaucoma increase with advancing age^17,18^, and the number of individuals with vision problems is anticipated to double by 2050^19–22^. The direct and indirect costs of managing visual impairment are a great public health challenge^22–25^. An individual with visual impairment and blindness is at risk of chronic health comorbidities, physical injuries, social withdrawal, and depression^12,22,24,25^. Moreover, cognitive function declines and visual impairment increases with increasing age^1,13^.

Despite the body of evidence, there are few reports regarding causal relationships or direct association between low vision and dementia. A clear understanding of this association may facilitate the development of strategies for reducing the burden of cognitive impairment. The National Health Insurance Service (NHIS) database has recently become accessible to researchers in Korea. This large-scale database permits the identification of the longitudinal incidence of diseases and allows for the analysis of the association between diseases and health conditions. Korea also has a unique rating system for disabled people, which involves grades for applicants as decided by the National Pension Service (Ministry of Health and Welfare of the Korean government), based on purely medical criteria. Using the six levels of disability, an individual with low vision can be classified depending on his/her visual acuity. Employing the disability grade used in Korea, we were able to investigate the impact of low vision on incident dementia by using a nationwide population-based cohort that includes over 1.5 million Koreans. We also performed a more detailed research question that includes what type of poor vision, the three different comparisons (overall dementia, specifically AD and VD), and interactions between vision and other risk factors.

## Results

### Baseline characteristics of the study subjects

The baseline clinical and biochemical characteristics of the subjects are summarized in Table 1. Among 6,029,657 subjects aged ≥40 years, 165,293 (2.74%), 123,497 (2.04%), and 20,678 (0.34%) were categorized into Dementia, Alzheimer’s disease (AD), and vascular dementia (VD) groups, respectively. Almost all parameters were significantly different between subjects with dementia, AD, and VD, and subjects without them (p < 0.05 almost all parameters excluding LDL for dementia and AD, income for VD); subjects with dementia, AD, and VD had less healthy statuses than subjects without dementia, AD, and VD. This may be because dementia is well known for age-related degenerative disease characteristics.

**Table 1:** Baseline Characteristics of the Study Subjects with or without Dementia.

	Dementia			Alzheimer’s disease			Vascular dementia		
	No	Yes	p	No	Yes	p	No	Yes	p
Number	5,864,364	165,293		5,906,160	123,497		6,008,889	20,768	
Sex (Male)	2,912,267(49.66)	62,416(37.76)	<0.0001	2,930,123(49.61)	44,560(36.08)	<0.0001	2,965,494(49.35)	9,189(44.25)	<0.0001
Place(urban)	2,650,947(45.2)	61,380(37.14)	<0.0001	2,666,359(45.15)	45,968(37.22)	<0.0001	2,704,641(45.01)	7,686(37.01)	<0.0001
Hypertension	1,914,059(32.64)	100,254(60.65)	<0.0001	1,940,015(32.85)	74,298(60.16)	<0.0001	2,001,056(33.3)	13,257(63.83)	<0.0001
Diabetes	658,059(11.22)	39,858(24.11)	<0.0001	668,690(11.32)	29,227(23.67)	<0.0001	692,508(11.52)	5,409(26.04)	<0.0001
Lipid	1,360,237(23.19)	54,473(32.96)	<0.0001	1,374,134(23.27)	40,576(32.86)	<0.0001	1,407,653(23.43)	7,057(33.98)	<0.0001
Exercise	2,977,729(50.78)	53,988(32.66)	<0.0001	2,991,895(50.66)	39,822(32.25)	<0.0001	3,024,541(50.33)	7,176(34.55)	<0.0001
Smoking			<0.0001			<0.0001			<0.0001
No	3,732,409(63.65)	12,5072(75.67)		3,762,488(63.7)	94,993(76.92)		3,842,910(63.95)	14,571(70.16)	
Past	924,976(15.77)	1,9151(11.59)		930,279(15.75)	13,848(11.21)		941,385(15.67)	2,742(13.2)	
Current	1,206,979(20.58)	21,070(12.75)		1,213,393(20.54)	14,656(11.87)		1,224,594(20.38)	3,455(16.64)	
Drinking level			<0.0001			<0.0001			<0.0001
No	3,420,258(58.32)	130,719(79.08)		3,451,919(58.45)	99,058(80.21)		3,535,516(58.84)	15,461(74.45)	
Mild	2,083,746(35.53)	28,785(17.41)		2,092,122(35.42)	20,409(16.53)		2,108,180(35.08)	4,351(20.95)	
Heavy	360,360(6.14)	5,789(3.5)		362,119(6.13)	4,030(3.26)		365,193(6.08)	956(4.6)	
Income (low)	1,523,999(25.99)	42,131(25.49)	<0.0001	1,534,720(25.99)	31,410(25.43)	<0.0001	1,560,821(25.98)	5,309(25.56)	0.1766
Age (Mean ± SD)	53.76 ± 10.12	71.56 ± 8.19	<0.0001	53.87 ± 10.21	72.04 ± 7.85	<0.0001	54.19 ± 10.45	69.19 ± 9.2	<0.0001
BMI	23.97 ± 3.03	23.61 ± 3.26	<0.0001	23.97 ± 3.03	23.58 ± 3.27	<0.0001	23.96 ± 3.04	23.85 ± 3.22	<0.0001
WC	81.06 ± 8.62	82.51 ± 8.67	<0.0001	81.07 ± 8.62	82.39 ± 8.66	<0.0001	81.09 ± 8.63	83.12 ± 8.61	<0.0001
Glucose	99.62 ± 24.25	105.71 ± 31.03	<0.0001	99.67 ± 24.32	105.38 ± 30.63	<0.0001	99.77 ± 24.44	107.08 ± 33.05	<0.0001
Systolic blood pressure	123.87 ± 15.27	129.88 ± 16.63	<0.0001	123.91 ± 15.29	129.74 ± 16.57	<0.0001	124.01 ± 15.33	130.84 ± 16.97	<0.0001
Diastolic blood pressure	77.05 ± 10.11	78.31 ± 10.25	<0.0001	77.06 ± 10.11	78.19 ± 10.18	<0.0001	77.08 ± 10.11	79.07 ± 10.49	<0.0001
HDL	54.78 ± 16.5	53.39 ± 20.64	<0.0001	54.77 ± 16.53	53.48 ± 20.93	<0.0001	54.75 ± 16.63	52.88 ± 18.71	<0.0001
LDL	117.17 ± 34.12	117.02 ± 36.59	0.0875	117.16 ± 34.13	117.33 ± 36.56	0.0919	117.17 ± 34.18	115.92 ± 36.48	<0.0001
Age (Median, range)	52 (46–60)	72 (68–77)	<0.0001	52 (46–61)	72 (68–78)	<0.0001	52 (46–62)	70 (64–76)	<0.0001
BMI	23.81 (21.89–25.82)	23.51 (21.37–25.67)	<0.0001	23.81 (21.89–25.82)	23.47 (21.36–25.64)	<0.0001	23.81 (21.88–25.82)	23.73 (21.64–25.91)	0.0124
WC	81 (75–87)	82 (77–88)	<0.0001	81 (75–87)	82 (76–88)	<0.0001	81 (75–87)	83 (77–89)	<0.0001
Glucose	95 (87–105)	98 (89–112)	<0.0001	95 (87–105)	98 (89–111)	<0.0001	95 (87–105)	98 (89–113)	<0.0001
Systolic blood pressure	121 (112–132)	130 (120–140)	<0.0001	121 (112–132)	130 (120–140)	<0.0001	122 (112–132)	130 (120–140)	<0.0001
Diastolic blood pressure	79 (70–82)	80 (70–84)	<0.0001	79 (70–82)	80 (70–84)	<0.0001	79 (70–82)	80 (70–85)	<0.0001
HDL	53 (45–63)	51 (43–61)	<0.0001	53 (45–62)	51 (43–61)	<0.0001	53 (45–62)	51 (43–60)	<0.0001
LDL	115 (94–138)	115 (92–140)	<0.0001	115 (94–138)	115 (92–140)	0.0672	115 (94–138)	114 (91–139)	<0.0001
TG*	117.19(117.13–117.24)	126.26(125.96–126.57)	<0.0001	117.26(117.21–117.31)	125.73(125.39–126.08)	<0.0001	117.39(117.34–117.44)	129.09 (128.21–129.98)	<0.0001

^*^Geometric means/Values are present as mean (SD) for continuous variables and n (%) for categorical variables.

BMI, Body mass index; WC, Waist circumference; HDL, High density lipoproteins; LDL, Low density lipoproteins; TG, Triglycerides

### Risk of dementia, Alzheimer’s disease, and vascular dementia according to severity of visual loss and visual impairment

**Figure 1:**
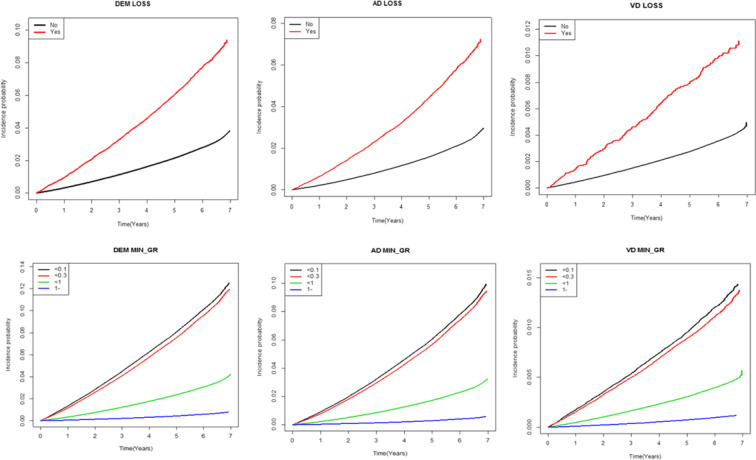
Kaplan-Meier cumulative probability curve for the incidence of dementia in patients with visual impairments. Dementia means overall dementia and is subcategorized as Alzheimer disease and vascular dementia. The top row (**A–C**) compares the subjects with visual loss with subjects without visual loss; subjects with visual loss show more increased incidence probability of dementia, AD, and VD than subjects without visual loss. The bottom row (**D–F**) compares subgroups according to visual impairments, and subjects with more severe visual impairments have more increased incidence probability of dementia, AD, and VD than subjects with less severe visual impairments.

Dementia, AD, and VD occurred more frequently in the visual loss and visual impairment group compared to the control group and this finding was statistically significant in both model 1 and model 2 (model 1: adjusted for age and sex, model 2: adjusted for age, sex, smoking, drinking, exercise, diabetes, hypertension, and lipid levels) (Table 2). For incidence rate per 1000 persons of dementia, AD and VD were significantly increased in patients who had higher visual loss grades (grade 1, 2, 3) and more severe visual impairments (best corrected visual acuity of the worse eye <0.1, <0.3) compared to patients who had lower visual loss grades (grade 4, 5, 6) and less severe visual impairments (best corrected visual acuity of worse eye <1.0, ≥1.0) (p < 0.01). Comparison between the subgroups according to severity of visual loss and visual impairments showed that subjects with more severe visual impairments have a higher risk of dementia, AD, and VD after adjusting for compounding variables; the hazard ratios (HR) of dementia increased significantly as visual acuity worsened (HRs 1.444 [95% CIs 1.415–1.473] for visual acuity (VA) < 1.0, 1.734 [1.693–1.777] for VA < 0.3, 1.727 [1.686–1.770] for VA < 0.1 and 1.991[1.902–2.085] for visual loss).We also analyzed the incidence of the probability of dementia, AD, and VD according to severity of visual loss and visual impairments. Figure 1 shows the Kaplan-Meier cumulative probability curve of the incidence probability of dementia. Incidence probability of dementia, AD, and VD increased over time in subjects who had visual loss and more severe visual impairments compared to subjects who did not have visual loss and had less severe visual impairments (Fig. 1).

**Table 2:** HRs (95% CI) for the development of dementia according to severity of visual impairments.

	Number	Dementia	Duration	IR per 1000	HR (95% C.I.)		AD	Duration	IR per 1000	HR (95% C.I.)	
					Model 1	Model 2				Model 1	Model 2
**Visual loss**											
No	5,999,693	163,077	34,517,933.23	4.7244	1(ref.)	1(ref.)	121,857	34,517,933.23	3.53025	1(ref.)	1(ref.)
Yes	29,964	2,216	166,914.84	13.2762	1.376(1.32,1.435)	1.346(1.29,1.403)	1,640	166,914.84	9.82537	1.356(1.291,1.424)	1.328(1.265,1.394)
**Visual impairment**											
<0.1	265,425	25,687	1,449,964.98	17.7156	1.792(1.75,1.836)	1.747(1.705,1.789)	19,512	1,449,964.98	13.4569	1.788(1.738,1.839)	1.747(1.698,1.797)
<0.3	238,594	22,000	1,315,615.03	16.7222	1.778(1.736,1.822)	1.739(1.698,1.782)	16,759	1,315,615.03	12.7385	1.783(1.733,1.835)	1.748(1.698,1.798)
<1.0	3,571,255	106,585	2,053,6251.49	5.1901	1.478(1.448,1.508)	1.445(1.416,1.475)	79,573	20,536,251.49	3.8748	1.503(1.467,1.54)	1.473(1.438,1.509)
≥1.0	1,954,383	11,021	1,138,3016.58	0.9682	1(ref.)	1(ref.)	7,653	11,383,016.58	0.6723	1(ref.)	1(ref.)
**Visual loss grade**											
No loss	5,999,693	163,077	34,517,933.23	4.7244	1(ref.)	1(ref.)	121,857	34,517,933.23	3.5303	1(ref.)	1(ref.)
Grade 4,5,6	23,868	1,549	134,111.89	11.5501	1.296(1.233,1.362)	1.274(1.211,1.339)	1,144	134,111.89	8.5302	1.279(1.207,1.356)	1.26(1.189,1.335)
Grade 1,2,3	6,096	667	32,802.95	20.3335	1.59(1.474,1.716)	1.535(1.423,1.657)	496	32,802.95	15.1206	1.561(1.429,1.705)	1.508(1.381,1.647)
**Visual loss and visual impairment**											
Loss	29,964	2,216	166,914.84	13.2762	2.08(1.986,2.178)	1.991(1.902,2.085)	1,640	166,914.84	9.8254	2.078(1.969,2.193)	1.997(1.892,2.107)
No loss & <0.1	243,272	23,905	1,326,864.38	18.0162	1.77(1.728,1.814)	1.727(1.686,1.77)	18,172	1,326,864.38	13.6954	1.763(1.714,1.815)	1.725(1.677,1.775)
No loss & <0.3	236,782	21,841	1,305,626.38	16.7284	1.773(1.73,1.817)	1.734(1.693,1.777)	16,646	1,305,626.38	12.7494	1.778(1.728,1.83)	1.743(1.694,1.794)
No loss & <1.0	3,566,526	106,330	20,509,741.78	5.1844	1.476(1.447,1.506)	1.444(1.415,1.473)	79,399	20,509,741.78	3.8713	1.501(1.466,1.538)	1.471(1.436,1.507)
No loss & ≥1.0	1,953,113	11,001	11,375,700.69	0.9671	1(ref.)	1(ref.)	7,640	11,375,700.69	0.6716	1(ref.)	1(ref.)
	**VD**	**Duration**	**IR per 1000**	**HR (95% C.I.)**							
				**Model 1**	**Model 2**						
**Visual loss**											
No	20,495	34,517,933.23	0.59375	1(ref.)	1(ref.)						
Yes	273	166,914.84	1.63556	1.399(1.241,1.576)	1.355(1.203,1.527)						
**Visual impairment**											
<0.1	2,905	1,449,964.98	2.0035	1.863(1.749,1.985)	1.792(1.682,1.909)						
<0.3	2,447	1,315,615.03	1.85997	1.791(1.679,1.912)	1.734(1.625,1.85)						
<1.0	13,585	20,536,251.49	0.66151	1.447(1.376,1.523)	1.401(1.332,1.474)						
≥1.0	1,831	11,383,016.58	0.16085	1(ref.)	1(ref.)						
**Visual loss grade**											
No loss	20,495	34,517,933.23	0.59375	1(ref.)	1(ref.)						
Grade 4,5,6	201	134,111.89	1.49875	1.368(1.19,1.572)	1.33(1.158,1.529)						
Grade 1,2,3	72	32,802.95	2.19492	1.497(1.188,1.886)	1.434(1.138,1.807)						
**Visual loss and visual impairment**											
Loss	273	166,914.84	1.63556	2.084(1.832,2.37)	1.957(1.721,2.225)						
No loss & <0.1	2,699	1,326,864.38	2.03412	1.855(1.739,1.979)	1.788(1.677,1.907)						
No loss & <0.3	2,425	1,305,626.38	1.85735	1.788(1.675,1.908)	1.731(1.622,1.847)						
No loss & <1.0	13,545	20,509,741.78	0.66042	1.447(1.375,1.523)	1.401(1.332,1.474)						
No loss & ≥1.0	1,826	11,375,700.69	0.16052	1(ref.)	1(ref.)						

NOTE. Model 1: adjusted for age, sex

Model 2: adjusted for age, sex, 3 levels of smoking and drinking, regular physical exercise, diabetes, hypertension, and lipid levels

Ref., reference. Incidence per 1,000 person-years. Bonferroni correction was used to control the overall significance level 0.05. Adjusted for age and sex. Adjusted for age, sex, smoking 3 levels, drinking 3 levels, regular physical exercise, diabetes, hypertension, and lipid levels.

### Risk of dementia in subjects with visual loss according to baseline parameters

We performed additional subgroup analysis of each type of adverse health event according to vision loss. Table 3 shows HRs (95% CI) of dementia in subjects with visual loss according to age, sex, smoking habits, drinking habits, exercise, diabetes, hypertension, and lipid levels, compared with subjects without visual loss. Subjects with visual loss, aged <65 years, or aged ≥65 years had a greater risk of developing dementia (HRs 1.550 for age <65 years and HRs 1.291 for age ≥65 years) and AD (HRs 1.537 for age <65 years and HRs 1.274 for age ≥65 years) than subjects without visual loss. There was a statistically significant interaction between age subgroups and the presence/absence of visual loss (p < 0.001 for dementia and p < 0.001 for AD). Subgroups with visual loss, no drinking habit, or mild drinking habit had a higher risk for dementia and AD than subgroups without visual loss. There was a statistically significant interaction between drinking habit and presence/absence of visual loss (p = 0.0091 for dementia and p = 0.0111 for AD). Subjects with visual loss, with diabetes or without diabetes were at a significantly greater risk of developing dementia than subjects without visual loss, and there was a significant interaction between the presence/absence of diabetes and the presence/absence of visual loss (p = 0.0323). Any other interactions of dementia with age, sex, exercise, hypertension, and lipid levels were not shown in the development of dementia, AD, and VD.

**Table 3:** HRs (95% CI) of dementia according to age, sex, smoking, drinking, physical exercise, diabetes, hypertension, and lipid levels.

	HR (95% C.I.)					
	Dementia	P for interaction	AD	P for interaction	VD	P for interaction
Age <65	1.55 (1.378,1.742)	<0.0001	1.537 (1.332,1.773)	<0.0001	1.536 (1.165,2.024)	0.0662
Age ≥ 65	1.291 (1.234,1.35)		1.274 (1.21,1.342)		1.302 (1.14,1.486)	
Male	1.304 (1.225,1.387)	0.3421	1.279 (1.189,1.377)	0.3417	1.289 (1.089,1.526)	0.3173
Female	1.373 (1.297,1.454)		1.362 (1.276,1.454)		1.437 (1.214,1.702)	
Smoking = non	1.31 (1.247,1.377)	0.1008	1.312 (1.239,1.389)	0.5505	1.259 (1.084,1.463)	0.1985
Smoking = past	1.433 (1.284,1.598)		1.359 (1.192,1.549)		1.684 (1.276,2.222)	
Smoking = current	1.431 (1.279,1.601)		1.374 (1.199,1.575)		1.465 (1.102,1.949)	
Drinking = non	1.33 (1.268,1.394)	0.0091	1.307 (1.237,1.381)	0.0111	1.405 (1.228,1.609)	0.269
Drinking = mild	1.455 (1.32,1.604)		1.464 (1.306,1.641)		1.298 (0.985,1.712)	
Drinking = moderate	1.047 (0.827,1.326)		1.03 (0.777,1.366)		0.782 (0.39,1.568)	
Physical exercise =non	1.338 (1.272,1.408)	0.4965	1.323 (1.248,1.403)	0.583	1.329 (1.148,1.537)	0.5924
Physical exercise=yes	1.337 (1.24,1.441)		1.314 (1.204,1.435)		1.405 (1.141,1.73)	
Diabetes = non	1.296 (1.232,1.363)	0.0323	1.289 (1.216,1.366)	0.1278	1.296 (1.118,1.503)	0.3845
Diabetes = yes	1.456 (1.351,1.57)		1.417 (1.297,1.549)		1.487 (1.214,1.822)	
Hypertension = non	1.353 (1.263,1.449)	0.0937	1.336 (1.234,1.446)	0.1737	1.5 (1.23,1.829)	0.0596
Hypertension = Yes	1.328 (1.259,1.4)		1.311 (1.232,1.395)		1.278 (1.101,1.485)	
Lipid = non	1.337 (1.27,1.407)	0.7436	1.319 (1.243,1.399)	0.8008	1.353 (1.167,1.567)	0.9892
Lipid = yes	1.363 (1.267,1.466)		1.346 (1.236,1.466)		1.362 (1.11,1.672)	

## Discussion

In this longitudinal nationwide study, we analyzed the risk of incident AD and VD according to visual function. Previously, there are very few studies that provide a large sample of adults with data on visual acuity and dementia incidence and diagnosis with consecutive follow-up data. The National Health Insurance System (NHIS) of Korea enables us to analyze a large dataset to examine and address the association between poor vision and dementia incidence. We also considered confounding factors including age, sex, smoking, drinking, exercise, diabetes, hypertension, and lipids levels. In addition, this study examines interactions between the covariates and poorer vision which adds a significant and important piece to address the various inconsistencies reported in the literature on vision and dementia and provides an overall picture on how other risk factors may interact with vision to influence cognitive impairment. After adjusting for confounding factors such as age, sex, smoking and drinking status, daily exercise, diabetes, hypertension, and dyslipidemia, we found that low vision increased the risk of overall dementia including AD and VD. The lower the vision of the individual, the higher the risk of dementia. Moreover, if low vision was present concurrently with risk factors, the risk of dementia was found to be higher.

Due to the increasing aging population, the prevalence of visual and cognitive impairment is increasing as well^4,6,24^; hearing and visual impairment is particularly common in older people with dementia^23,24^. Previous large-scale studies showed the association between visual impairment and cognitive function. The Singapore Malay Eye study, which included 1179 participants aged 60 to 80 years, showed that persons with visual impairment were more likely to have cognitive dysfunction after adjusting for age, sex, education level, income, and type of housing^12^. Using a logMAR visual acuity chart to measure visual acuity and a locally validated Abbreviated Mental Test to define cognitive dysfunction, the researchers found that those with visual impairment, particularly due to cataract, were more likely to have cognitive dysfunction. The Singapore Malay study, which was a population-based cross-sectional study had a limitation of using a lower specificity tool to define cognitive function. The Age-Related Eye Disease Study (AREDS) also suggested a possible association of advanced age-related macular degeneration and visual acuity with cognitive impairment in older persons^8^. Out of the available AREDS subjects, 2946 were included in the study, and increased macular abnormalities were shown to reduce mean cognitive function scores as measured with the Modified Mini-Mental State Examination and the Wechsler Logical Memory Scale. In another study that used data from the National Health and Nutrition Examination Survey (NHANES), and the National Health and Aging Trends Study (NHATS) in US adults, vision at distance and based on self-reports was found to be significantly associated with worse cognitive function after adjusting for demographics, health, and other factors^24^. In a national survey in the UK, significant visual impairment (VA worse than 6/12) was 32.5%, and the prevalence was disproportionately higher in people with dementia living in care homes^11^. Many of these studies focused on under-diagnosed and under-treated visual impairment in the dementia population. Despite many evidences that visual and cognitive impairment are related, a common intrinsic limitation of the previous cross-sectional reports is that those results could not determine whether visual impairment influences the rate of cognitive decline. The primary endpoint of our study was newly diagnosed dementia (AD and VD); therefore, patients who had dementia that was diagnosed prior to enrolment were excluded. Thus, the present study revealed that having disability and low vision is significantly associated with development of both AD and VD.

With the respect to the impact of other factors on dementia, we categorized the study subjects according to their ages, sex, lifestyle (smoking, drinking, and exercise), and metabolic status (diabetes, hypertension and dyslipidemia), and analyzed the hazard ratio after controlling confounding factors. We found that the risk of dementia was significantly higher in subjects with vision loss, who were aged less than 65 years, had a diabetes diagnosis, and who reported no or mild alcohol intake. The population younger than 65 years showed over 50% increased risk of incident dementia if the individual had a disability rating. On the contrary, the older population aged over 65 showed 29.1%, 27.4%, and 30.2% increased risk of dementia, AD, and VD, respectively, which was lower compared to the younger population. Since the most common causes of low vision may be cataract, AMD, diabetic retinopathy, and glaucoma^1,2^, the relatively younger subjects who have low vision and disability are prone to have systemic comorbidities and these factors could affect cognitive function as well. Subjects with diabetes had a higher HR of dementia, AD, and VD if the subjects had comorbid visual disabilities. We surmised that an individual with vision loss may have difficulty in controlling diabetes due to physical limitations and challenges in attending hospital visits. This may lead to diabetes-related complications of which dementia is suggested to be one of those.

Although a lot of progress has been made over the past decade, controversies remain regarding the pathogenesis of AD and VD^13,14,16^. Majority of AD occurs on an apparently sporadic basis and typical late onset AD is likely to be driven by a complex interplay between genetic and environmental factors^13,14^. In addition to age, risk factors for Alzheimer’s disease include vascular risk factors such as hypertension, atrial fibrillation, obesity, diabetes, ischemic heart disease, hypercholesterolemia, and other factors such as homocysteine concentrations^16,26–29^. Subcortical vascular disease as well as cortical infarcts attribute to VD, and the risk factors are advancing age, vascular risk, and low education; the female sex is also suggested to increase the risk of VD^16,26,27,29^. Interestingly, in AMD, retinal neurons are the major site of pathogenesis, and retinal disruption is the cause of vision loss^8,23^. Moreover, the amyloid β, which is one of the core cerebrospinal biomarkers for AD, is found in drusenoid deposits in AD patients^14,30,31^. In this context, AMD may be a neurodegenerative disease and may share a common pathogenic mechanism with AD.

The main strength of this study is that the data used was from a large-scale nationwide database that represents the entire Korean population. Where other studies mostly focused on the older population, we included not only the aged population but the middle-aged population (between 40 to 60 years old) as well, to analyze the association of low vision as well as aging itself with dementia. Our data suggest that visual impairment is a significant risk factor in the middle-aged and older populations. However, our study has several limitations that should be noted. Firstly, there may be a discordance between the actual number of subjects that make up the dementia population and the information of claim data. We tried to reduce the error by combining the diagnosis statements and prescription statements. Secondly, the severity of cognitive impairment was not assessed. If there is an association between visual acuity and severity of cognitive dysfunction, the causal relationship could be discussed. Additionally, since recognition functions other that vision, such as hearing loss, have received some attention for their role in the development of dementia^32^, more detailed analysis of general cognitive impairments needs to be performed. Thirdly, the disability grade from 1 to 6 could be due to factors other than visual impairment. In the Korean disability rating system, we could only find the grade and not the specific parameters of the grading. If the individual has good visual acuity and disability in other part of the body, he or she could be a source of bias in the data analysis. To overcome this issue, we classified the study subjects further based on disability grading and visual acuity.

In conclusion, individuals with low visual acuity have increased risks of dementia. The medical costs of these dementia are projected to almost double over the next 10 years. Therefore, attention to associated risk factors is important in terms of general health. The results of this study suggest that low visual acuity is an independent risk factor of dementia that must be considered when preparing health policies.

## Materials and Methods

### Study population

The National Health Insurance System (NHIS) of Korea uses data generated from two major sources: National Health Insurance (NHI), which covers 97% of the population, and Medical Aid (MA), which covers the remaining 3% of the population who have low income^33,34^. All Koreans have to be registered in these two programs. This database includes demographic information (e.g., age, sex, income), claim information (diagnosis defined by International Classification of Disease (ICD) codes and details of prescriptions), and health checkup information (results of basic laboratory tests and questionnaires about past medical history, current medications, and life style). In this study, we used the NHIS database, a well-confirmed cohort that represents the entire Korean population, which is over 50 million people.

**Figure 2:**
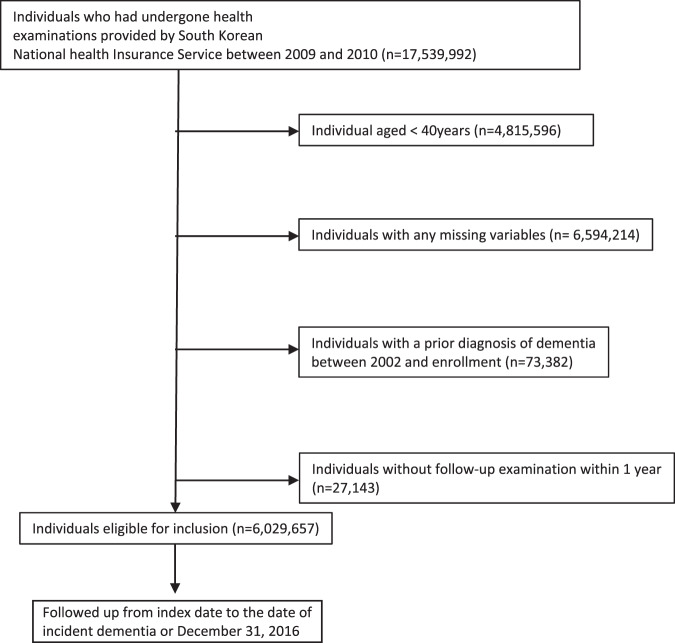
Enrolment flowchart for the study population.

We included subjects who underwent health examinations between January 1, 2009 and December 31, 2010 (index period). Out of the 17,539,992 subjects, those who had missing baseline data (n = 6,594,214), age <40 years (n = 4,815,596), those who received a diagnosis before the index period (n = 73,382), and those who went without follow-up examination within 1 year (n = 27,143) were excluded (Fig. 2). A total of 6,029,657 adults were included in the study and were followed up for 5.75 ± 0.92 years (mean ± SD). This study was approved by the Institutional Review Board of the Catholic University of Korea, and the need for informed consent for study was waived as part of the study approval.

### Clinical and laboratory measurements

To generate the health examination data, all subjects completed a questionnaire on their medical history, use of tobacco and alcohol, and exercise habits. Smoking was classified as non-current or current, alcohol consumption was categorized as mild drinker (<30 g per day) or heavy drinker (≥30 g per day), and exercise level was classified as less than three times per week or moderate to vigorous exercise three or more times per week (physically active). We defined low socioeconomic status as income in the lowest 20%. Body mass index (BMI) was calculated as body weight (kg) divided by the square of the height (m^2^). Blood pressure (BP) was measured with the subject in a sitting position after five minutes of rest. After overnight fasting, blood samples were collected. Serum glucose, total cholesterol, triglyceride (TG), and high-density lipoprotein levels were also evaluated. Baseline comorbidities were identified as follows: hypertension (treatment with antihypertensive medication or systolic/diastolic blood pressure ≥140/90) and diabetes mellitus (DM) (treatment with antidiabetic drug or fasting glucose level ≥126 mg/dL).

### Definition of incident dementia

Incident dementia and prescription of anti-dementia medication at the same time was considered a diagnosis of dementia (ICD-10 codes F00, G30, F01, F02, F03, G23.1, G31.0, G31.1, G31.82, G31.83, G31.88, and F10.7) (Table 4). The anti-dementia medications include acetylcholinesterase inhibitors (rigastigmine, galantamine, or donepezil) or N-methyl-D-aspartate antagonist (memantine). Patients with dementia were grouped into either the AD (ICD-10 codes F00 and G30) or the VD (ICD-10 code F01) group based on the diagnosis code at the first visit. If diagnoses of both AD and VD were recorded at the first visit, we used the diagnosis of the second visit as the final diagnosis. If the main diagnosis was neither AD nor VD, the dementia was defined as “other dementia”^26,35^.

**Table 4:** ICD-10 codes for various types of dementia.

F00	Dementia in Alzhemier’s disease
G30	Alzhemier’s disease
F01	Vascular dementia
F02	Dementia in other disesase classified elsewhere
F03	Unspecified dementia
G23.1	Progressive supranuclear ophthalmoplegia
G31.0	Circumscribed brain atrophy
G31.1	Senile degeneration of brain, NEC
G31.82	Dementia with Lewy bodies
G31.83	Corticobasal syndrome
G31.88	Other specified degenerative disease of nervous system
F10.7	Residual and late onset psychotic disorder due to use of alcohol

### Definition of vision loss and visual impairment

Visual acuity was measured at the initial screening. Vision loss was defined as having a visual disability grade, which is assigned based on national designation criteria (referred by Table 5). Subjects with visual disability included individuals with certifications of visual disability from the Korean government (Ministry of Health and Welfare), which is based on the medical records and documents from a certificated ophthalmologist. Visual impairment was classified as having the best corrected visual acuity of the worse eye <0.1, <0.3, <1.0, or ≥1.0. Visual disability grades 4, 5, and 6 mean less severe visual disability than grades 1, 2, and 3. In other words, the subjects with visual disability grade 1 means that the subjects have most severe visual impairments. Table 5 describes detailed criteria in visual disability grades.

**Table 5:** Criteria for grading visual disability as determined by the National Pension Service (Ministry of Health and Welfare).

Grade 1	Visual acuity of better eye is less than or equal to 0.02
Grade 2	Visual acuity of better eye is less than or equal to 0.04
Grade 3	Visual acuity of better eye is less than or equal to 0.06, or the visual field of each eye is less than 5 degrees in any direction
Grade 4	Visual acuity of better eye is less than or equal to 0.1, or the visual field of each eye is less than 10 degrees in any direction
Grade 5	Visual acuity of better eye is less than or equal to 0.2, or the sum of visual fields of both eyes is less than 50% of normal
Grade 6	Visual acuity of worse eye is less than or equal to 0.02

### Study protocol approvals and registrations

The Deliberative Committee of the Health Insurance Review and Assessment service (HIRA) approved the conditional use of the database of this study. The study adhered to the tenets of the Declaration of Helsinki, and the study protocol was reviewed and approved by the Institutional Review Board of Catholic University of Korea (SC19ZESE0061).

### Statistical analysis

Statistical analyses were conducted using SAS 9.4 software (SAS institute, Cary, NC). Clinical and demographic characteristics of the study subjects are presented as mean ± SD for continuous variables, or number (percentage) for categorical variables. For each participant, the primary outcome between January 1, 2009 and December, 31 2010 was dementia, and the number of person-years follow-up was counted. Dementia, AD, and VD were assessed as incident dementia during 5.75 ± 0.92 years after the last recorded check-up. Incidence of dementia was calculated by dividing the number of events by 1,000 person-years^32^. Cox proportional hazards regression models were performed to evaluate the association of visual loss and visual impairments with incident dementia^36,37^; Bonferroni-adjusted 95% CIs were calculated as well. Model 1 was adjusted for age and sex, and Model 2 was adjusted for age, sex, smoking, drinking 3 levels, regular exercise, diabetes, hypertension, and lipid levels. Stratified analyses were performed according to age, sex, smoking 3 levels, drinking 3 levels, exercise, diabetes, hypertension, and lipid level, and interactions between subgroups were evaluated. Two tailed *P* values were analyzed and *P* < 0.05 was considered statistically significant.

## Data Availability

The datasets generated during and/or analyzed during the present study are available from the corresponding author on reasonable request.
